# De Novo Mutation in *TMEM151A* and Paroxysmal Kinesigenic Dyskinesia

**DOI:** 10.1002/mds.29023

**Published:** 2022-05-19

**Authors:** Thomas Wirth, Aurélie Méneret, Nathalie Drouot, Gabrielle Rudolf, Ouhaid Lagha Boukbiza, Jamel Chelly, Christine Tranchant, Amélie Piton, Emmanuel Roze, Mathieu Anheim

**Affiliations:** ^1^ Service de Neurologie Hôpitaux Universitaires de Strasbourg Strasbourg France; ^2^ Institut de Génétique et de Biologie Moléculaire et Cellulaire Institut National de la Santé Et de la Recherche Médicale‐U964/Centre National de la Recherche Scientifique‐UMR7104/Université de Strasbourg Illkirch‐Graffenstaden France; ^3^ Fédération de Médecine Translationnelle de Strasbourg Université de Strasbourg Strasbourg France; ^4^ Département de neurologie, Assistance Publique Hôpitaux de Paris Hôpital Pitié‐Salpêtrière Paris France; ^5^ Sorbonne Université, Institut du Cerveau, Institut National de la Santé Et de la Recherche Médicale‐U1127/Centre National de la Recherche Scientifique‐UMR7225 Salpêtrière Hospital, AP‐HP Paris France; ^6^ Laboratoire de Diagnostic Génétique Hôpitaux Universitaires de Strasbourg Strasbourg France

We read with great interest the article by Tian and colleagues.^1^ Heterozygous mutations in *TMEM151A*, encoding transmembrane protein 151 A, a protein of undetermined function, have been very recently associated with paroxysmal kinesigenic dyskinesia (PKD) in the Chinese population.^1^, ^2^, ^3^, ^4^ *TMEM151A* is highly expressed in the brain, including the cerebral cortex and the thalamus and is highly conserved among species. To definitively confirm the association between *TMEM151A* and PKD, other mutations in the same gene should be identified in independent cohorts from different populations.

We applied whole exome sequencing (WES) on 23 French patients with sporadic PKD who tested negative for *PRRT2* (proline‐rich transmembrane protein 2) mutations as well as their asymptomatic parents. PKD diagnosis was made by movement disorders specialists according to the consensus clinical criteria.^5^ WES, bioinformatic analysis, and variant prioritization were performed as previously described.^6^ Variants were classified according to the American College of Human Genetics and Genomics (ACMG) criteria.^7^ All patients gave written informed consent before genetic testing, and a local ethics committee approved the study. We identified a de novo missense variant (c.166G > C [p.Gly56Arg]) in *TMEM151A* in a single patient (Fig. 1) through trio‐based exome sequencing. This variant, absent from public databases including Exac, 1000G, and GnomAD, led to a substitution in the second transmembrane domain of the protein near previously reported pathogenic variants, such as c.140 T > C [p.Leu47Pro] or c.133 T > G [p.Cys45Arg]. It was predicted to be damaging by Polyphen, with a Combined Annotation Dependent Depletion (CADD) score above 20. The phenotype was consistent with previous reports of *TMEM151A*‐related PKD. The patient had no history of infantile seizures and presented with brief attacks of dystonia triggered by voluntary movements, surprise, or stressful events beginning after age 16. Before medication initiation, the patient experienced between 10 and 20 attacks a day, usually lasting a few dozens of seconds. Attacks could be focal or generalized, affecting speech or involving the face or upper and lower limbs subsequently or simultaneously. The attacks totally ceased after the initiation of low doses of lamotrigine (50 mg/d). This variant was subsequently classified as likely pathogenic (class IV) according to the ACMG criteria (PS2 + PM1 + PM2 + PP2 + PP4).^7^ No other de novo class IV or V variant was identified in this cohort. The *TMEM151A* mutation was identified in one of 23 patients of our *PRRT2*‐negative PKD cohort, which is in accordance with the frequency of 4.8% found by Tian et al.^1^

**FIG 1:**
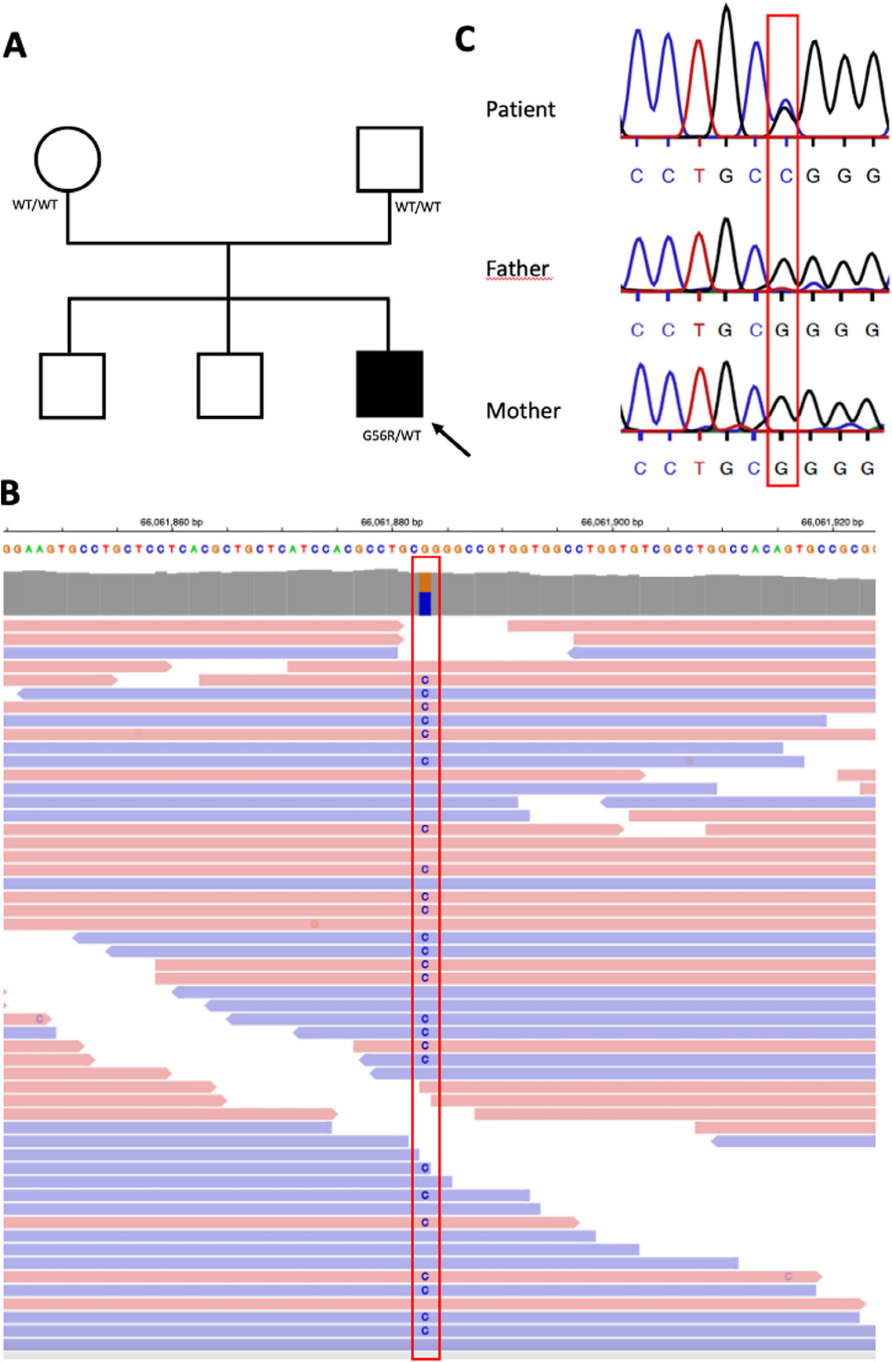
(**A**) Pedigree of the family. WT: Wild Type (**B**) Visualization of the (c.166G > C [p.Gly56Arg]) variant (between red lines) in the patient in the *TMEM151A* (Transmembrane protein 151 A) sequence through the Integrated Genome Viewer. The replacement of the reference G by a C (blue) is present on half of the patient's read, meaning heterozygosity. (**C**) Sanger sequencing of the (c.166G > C [p.Gly56Arg]) variant (between red lines). The variant is present in a heterozygous state in the patient but absent in the two asymptomatic parents, compatible with a de novo occurrence. [Color figure can be viewed at wileyonlinelibrary.com]

We report on a de novo mutation in *TMEM151A* in a patient with PKD. Our findings confirm *TMEM151A* variants as a genetic cause of PKD and suggest that de novo mutations in this gene are infrequently responsible for sporadic PKD cases.^4^ Further works are warranted to refine the phenotype/genotype correlations among *TMEM151A*‐related disorders. Whether *TMEM151A* is a transmembrane protein involved in synaptic function and whether *TMEM151A*‐related PKD is underpinned by its loss of function also remain to be elucidated.

## Author Roles

(1) Research Project: A. Conception, B. Organization, C. Execution; (2) Statistical Analysis: A. Design, B. Execution, C. Review and Critique; (3) Manuscript: A. Writing First Draft, B. Review and Critique.

T.W.: 1A, 1C, 2C, 3A

A.M.: 1A, 1C, 2C, 3B

N.D.: 1C, 2C

G.R.: 1C, 2C

O.L.B.: 1C

J.C.: 1A, 1C, 3B

C.T.: 1A, 1C, 3B

A.P.: 1A, 1C, 3B

E.R.: 1A, 1B, 1C, 3B

M.A.: 1A, 1B, 1C, 2C, 3B

## Financial Disclosures

Thomas Wirth received grants from the Revue Neurologique, the Fondation Planiol, and the Association des Personnes Concernées par le Tremblement Essentiel (APTES) organizations and travel funding from LVL Medical. Aurélie Méneret received speaker honoraria from Abbvie. Emmanuel Roze received honorarium from Orkyn, Aguettant, and Elivie for speeches and for participating in the advisory board of Allergan and received research support from Merz‐Pharma, Orkyn, Aguettant, Elivie, Ipsen, Allergan, Everpharma, Fondation Desmarest, Association des Malades Atteints de Dystonie (AMADYS), ADCY5.org, Agence Nationale de la Recherche, Societé Française de Médecine Esthétique, and the Dystonia Medical Research Foundation. The other authors declare no competing interest.

## Data Availability

Anonymized data pertaining to the research presented will be made available upon reasonable request from external investigators.
